# Genetic structure and differentiation from early bronze age in the mediterranean island of sicily: Insights from ancient mitochondrial genomes

**DOI:** 10.3389/fgene.2022.945227

**Published:** 2022-09-09

**Authors:** Alessandra Modi, Maria Teresa Vizzari, Giulio Catalano, Rajiv Boscolo Agostini, Stefania Vai, Martina Lari, Chiara Vergata, Valentina Zaro, Lucia Liccioli, Mariaelena Fedi, Serena Barone, Lorenzo Nigro, Hovirag Lancioni, Alessandro Achilli, Luca Sineo, David Caramelli, Silvia Ghirotto

**Affiliations:** ^1^ Department of Biology, University of Florence, Firenze, Italy; ^2^ Department of Life Sciences and Biotechnology, University of Ferrara, Ferrara, Italy; ^3^ Department of Biological, Chemical and Pharmaceutical Sciences and Technologies, University of Palermo, Palermo, Italy; ^4^ INFN (Istituto Nazionale di Fisica Nucleare) Sezione di Firenze, Firenze, Italy; ^5^ Department of Physics and Astronomy, University of Florence, Florence, Italy; ^6^ Department “Italian Institute of Oriental Studies—ISO”, Sapienza University of Rome, Rome, Italy; ^7^ Department of Chemistry, Biology and Biotechnology, University of Perugia, Perugia, Italy; ^8^ Department of Biology and Biotechnology “L. Spallanzani”, University of Pavia, Pavia, Italy

**Keywords:** ANCIENT DNA, mitochondrial genomes, genetic structure, coalescent simulations, approximate bayesian computation

## Abstract

Sicily is one of the main islands of the Mediterranean Sea, and it is characterized by a variety of archaeological records, material culture and traditions, reflecting the history of migrations and populations’ interaction since its first colonization, during the Paleolithic. These deep and complex demographic and cultural dynamics should have affected the genomic landscape of Sicily at different levels; however, the relative impact of these migrations on the genomic structure and differentiation within the island remains largely unknown. The available Sicilian modern genetic data gave a picture of the current genetic structure, but the paucity of ancient data did not allow so far to make predictions about the level of historical variation. In this work, we sequenced and analyzed the complete mitochondrial genomes of 36 individuals from five different locations in Sicily, spanning from Early Bronze Age to Iron Age, and with different cultural backgrounds. The comparison with coeval groups from the Mediterranean Basin highlighted structured genetic variation in Sicily since Early Bronze Age, thus supporting a demic impact of the cultural transitions within the Island. Explicit model testing through Approximate Bayesian Computation allowed us to make predictions about the origin of Sicanians, one of the three indigenous peoples of Sicily, whose foreign origin from Spain, historically attributed, was not confirmed by our analysis of genetic data. Sicilian modern mitochondrial data show a different, more homogeneous, genetic composition, calling for a recent genetic replacement in the Island of pre-Iron Age populations, that should be further investigated.

## Introduction

Sicily is the largest island of the Mediterranean Sea, and it is widely known for its key role as a crossroad for human colonization from the early Holocene to the Middle Age. Indirect evidence of human presence in Sicily, based on lithic surface finds (Bianchini, 1972, 1971, 1969), could suggest an early settlement, but the evidence is insufficient and therefore it is thought that the human presence in the island does not pre-date the Last Glacial Maximum (Sineo et al., 2015) (LGM, for a commentary Sineo et al., 2015); however, it is only from the Late Glacial—Epigravettian period that a clear large-scale human presence in the island is documented, with the oldest human remains dated about 14,000 cal BCE (Addaura 1, Mannino et al., 2011). Since the middle Holocene, Sicily was settled by Neolithic Farmers from Anatolia and the Near East, as documented by the presence of Impressed Ware (Stentinello culture) dated about 5,800–5,500 cal BCE (Pessina and Tiné, 2008; Natali and Forgia, 2018). Historical and archaeological data offered a detailed description of more recent population settlements within the island, as the Italic people from Italian peninsula, the Phoenicians, the Greeks, the Romans, the Byzantines, the Arab and the Normans (Finley, 1968; Romano et al., 2021). In the pre-Hellenistic period, three distinct groups of people occupied the island: the Sicels (in the East), the Sicanians (in the West) and the Elymians (in the extreme West). The Sicels spoke an Indo-European dialect of the Italic group, as the Elymians, although ascribed to Trojan origin (Finley, 1968). No trace of the Sicanian language remains, and also their origin is extremely controversial. The Athenian historian Thucydides (VI, 2, 2) indeed postulated they came from Iberia (Finley 1968), while other authors proposed a local origin (Moscati, 1980). Toward the end of the second millennium BCE, Sicily was reached by Phoenician settlers who journeyed from Lebanon across the Mediterranean and established trading stations on promontories and on small islands around the coast. When the Greek colonization began (around the eighth century BCE) the Phoenicians withdrew toward the western corner of the island, possibly causing internal east-west cultural differentiation subsequently reshaped by later conquerors (Byzantines, Islamic and Normans) (Sarno et al., 2017).

Complex demographic and cultural dynamics should have affected the genomic landscape of Sicily at different levels; however, the geographical origin of some of the cultures dwelling in the Island, as well as the relative impact of these migrations on the genomic structure and differentiation of current Sicilian populations is actually largely unknown. The available genetic data are indeed mainly from modern populations, both uniparental (mitochondrial DNA and Y chromosome) (di Gaetano et al., 2009; Romano et al., 2003; Sarno et al., 2014) and autosomal (SNPs; Sarno et al., 2017), and generally only show that present-day Sicilian populations are characterized by shared genetic affinity with the modern inhabitants of Southern Italy and a large portion of eastern Mediterranean shores, have a predominant Neolithic-like component, and a substantial homogenous composition of paternal and maternal genetic pools. When analyzing modern genetic data, however, our ability to infer the genetic impact of different past migration processes is challenged by the number of demographic events and admixture layers involving ancestral populations, and by the level of genetic affinity among the putative sources of admixture. In this case, direct genomic evidence from key ancient populations would hence be fundamental to successfully quantify the historical presence of genetic structure, and to assess the genetic impact of past population dynamics and of the complex colonization histories. Unfortunately, the amount of ancient Sicilian genetic data is limited and scattered over time periods. Up to date, paleogenetics studies have focused mainly on the first colonization of the Island. Recently, the complete mitochondrial genome from Upper Paleolithic San Teodoro two and Mesolithic Oriente B individuals (Modi et al., 2021, 2020) revealed a low genetic diversity of the Sicilian hunter-gatherers presumably because of genetic drift processes during the early stages of colonization. Genome wide data from Late Epigravettian Oriente C individual (Mathieson et al., 2018; Catalano et al., 2020) confirmed the Western hunter-gatherer (WHG) affiliation of the specimen, suggesting a substantial genetic homogeneity among the Mediterranean Epigravettian population. Other whole-genome SNPs from ancient Sicilian individuals from Middle-Neolithic to Late Bronze Age has been recently produced (Fernandes et al., 2020), but the analyses presented in the paper were specifically conducted to extract information about the impact of the Bronze Age migration from Steppe in the islands of Western Mediterranean. Diroma et al., 2021 produced 27 complete mitochondrial sequences from an Iron Age necropolis in Polizzello but did not perform a comparison with other ancient Sicilian genomes, nor with other continental populations.

**FIGURE 1:**
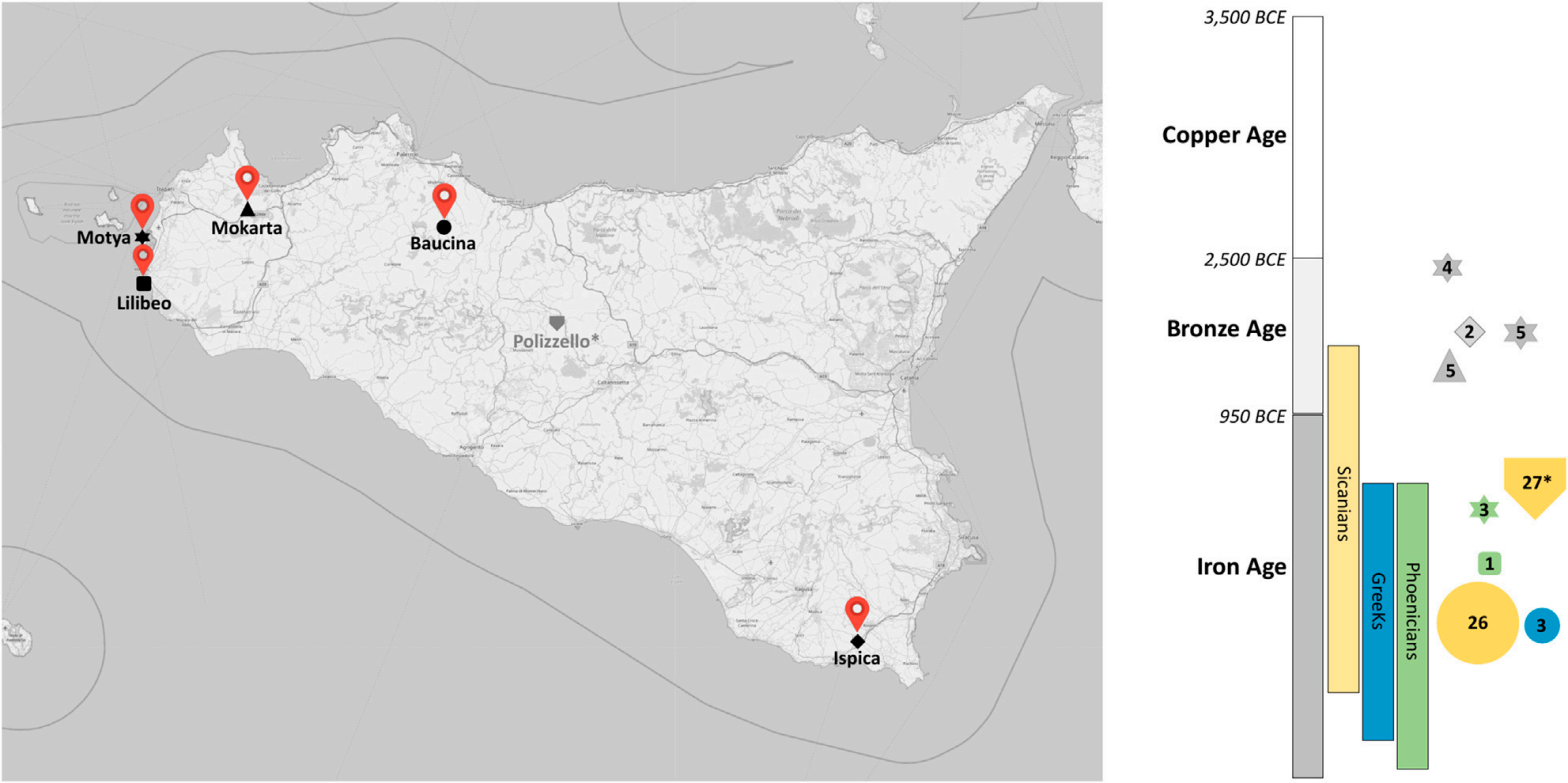
Samples geographical and chronological distribution. Map of Sicily showing the location of the archaeological sites from which individuals were sampled. Time period and civilizations covered in this study are shown by color blocks with collected samples represented on the right side. The size of the symbols is proportional to the number of individuals. *Samples from Diroma et al., 2021.

In this work, we reconstructed 43 complete or nearly complete mitochondrial genomes from individuals retrieved in five necropolises located in the island of Motya (western coast of Sicily) and in the archaeological sites of Baucina, Mokarta, Lilibeo and Ispica (see Figure 1), with the final aim of exploring the ancestral genetic structure of the Island and contextualizing this variation with that present in coeval groups from the Mediterranean area, as well as with that of its current inhabitants. To this scope we combined the newly generated sequences with other Sicilian mitochondrial genomes available from literature (Fernandes et al., 2020; Diroma et al., 2021), and with an extended dataset of about 300 complete mitochondrial sequences from the Mediterranean basin (Supplementary Table S1). To make up for the lack of unbiased modern mito-genetic data from Sicily, often focusing on specific macro-haplogroups and hence not being representative of the population’s genetic variability, we obtained a new mito-haplogroup assignment from 175 modern individuals. These samples come from all extant administrative provinces in Sicily, and may be properly compared with the haplo group variation retrieved in the ancient samples. Our results indicate a notable genetic structure within Sicily since the Early Bronze Age, in agreement with the complex historical cultural background of the Island. Approximate Bayesian Computation (ABC) model testing further allowed us to make explicit predictions about the origin of one of the most impressive cultures of Sicily, the Sicanian culture. Finally, the comparison with modern data advocates for a recent homogenization of the genetic variation, possibly resulting from a recent genetic turnover of pre Iron-Age populations whose dynamics should be further investigated.

## Materials and methods

### Sequencing of mitochondrial genomes, bioinformatic analysis, and dating

Ancient sample preparation and sequencing. We collected petrous bones and teeth of 58 individuals from five different archeological sites: 16 specimens from Motya, 34 from Baucina, five from Mokarta, one from Lilibeo and two from Ispica, spanning from the Bronze Age to the Hellenistic period (Figure 1 and Supplementary Table S2). For Baucina, we analyzed samples from two cultural contexts classified on the basis of the burial rites: 31 Sicanian and three Greeks (Belvedere et al., 2017). Sampling location and archaeological context of the samples we generated are detailed in Supplementary Data. Molecular analysis was carried out at the Laboratory of Anthropology (University of Florence) in exclusively dedicated ancient DNA facilities and following strict guidelines to avoid contaminations (Gilbert et al., 2005; Willerslev and Cooper, 2005). Moreover, blank controls were included during all DNA extraction and library preparation experiments to monitor for contamination of the reagents. Before powder sampling, the outer layer of temporal bones and teeth was brushed with disposable tools and irradiated by ultraviolet light (254 nm) for 30 min to remove external contaminants. For petrous bones, powder was collected from the densest part of the inner ear as described in Pinhasi et al., 2015 using a dentist microdrill with disposable tips. For teeth, powder was recovered from the inner dentine in root canals, taking care to preserve the crown morphology. For each sample, approximately 50 mg of bone or dentine powder were used for DNA extraction, applying a protocol optimized to retain short DNA fragments (Dabney et al., 2013). A double-strand and double indexed library was generated from 20 µL of each extract (Meyer and Kircher, 2010). No uracil-DNA glycosylase (UDG) treatment was performed for 29 libraries of Baucina samples, while all the other libraries were processed with partial UDG treatment (Rohland et al., 2015) for reducing the rate of deamination (Supplementary Table S2). Each library was then enriched for mtDNA following a multiplexed capture protocol as described in Maricic et al., 2010. After quality control, libraries were sequenced on Illumina MiSeq in paired-end mode, setting 2 × 75 + 8 + 8 run parameters.

Mitochondrial consensus sequences reconstruction and contamination estimation. Sequencing data were demultiplexed and sorted according to the individual sample barcodes added to each DNA fragment during library preparation. Raw reads were then processed using the pipeline described in Peltzer et al., 2016. Adapters were trimmed and reads with a minimum overlap of 10 bp were merged in a single sequence using Clip & Merge v1.7.4. Merged reads were mapped on the revised Cambridge Reference Sequence (rCRS, NC_012920) using CircularMapper and BWA v.0.6.2 (Li and Durbin, 2009), setting -l 16,500 -o two and -n 0.01 parameters to optimize alignment performance with degraded DNA (Schubert et al., 2012). Reads with mapping quality below 30 were discarded. PCR duplicates were removed using DeDup v0.12.1, a tool that takes both mapping positions (start and end) into account to identify duplicates. Degradation patterns (i.e fragment length and deamination rates at the ends of the molecules) were estimated using MapDamage 2.0 (Jónsson et al., 2013). Schmutzi software (Renaud et al., 2015) was used for endogenous consensus calling and for estimating present-day human contamination (details in Supplementary Data). Bases with individual likelihood <20 were considered as missing positions (Ns) in the reconstructed mitogenome sequences (see Supplementary Tables S3, S4). For each sample, mitochondrial haplogroup was determined with Haplogrep2 (van Oven, 2015; Weissensteiner et al., 2016) on the command line, using fasta file as input.

Radiocarbon dating. Radiocarbon measurements were performed on collagen extracted from petrous bones according to the modified-Longin method. After reducing to graphite the collected material, C-14 concentration in unknown samples was measured by Accelerator Mass Spectrometry (Fedi et al., 2007). Conventional radiocarbon ages were converted to calibrated ages using OxCal 4.3 by the intCal13 calibration curve (Reimer et al., 2013).

Data collection and processing. We collected 300 high quality complete mitochondrial genomes from the literature accessible on public databases such as The European Nucleotide Archive (ENA, https://www.ebi.ac.uk/ena) and GenBank (https://www.ncbi.nlm.nih.gov/genbank). The samples from Fernandes et al. (2020) were mapped to the reconstructed human mtDNA consensus sequence (RSRS, Behar et al., 2012) so we decided to re-align these sequences to the Revised Cambridge Reference Sequence (rCRS, GenBank Accession Number NC_012920) in order to make them comparable to the other collected sequences. We mapped the raw reads using BWA-aln v.0.7.15 (Li and Durbin, 2009), with parameters -n 0.01 and, -o 2 -L 16,500 and used picard-tools-1.98 (http://picard.sourceforge.net/) to add read groups (AddOrReplaceReadGroups) and to remove duplicated reads from the obtained BAM files (MarkDuplicates).

Reads showing a mapping quality below 30 were filtered out from all the alignment files collected in our datasets. We then used the schmutzi pipeline described above to estimate the endogenous mitochondrial consensus sequence for each sample.

Finally, we performed a multiple sequence alignment of our dataset exploiting the software MAFFT (Katoh and Standley, 2013) with the following options FFT-NS-2 and-maxiterate 100.

Modern dataset. The modern collection consisted of 175 biological samples from unrelated subjects with a Sicilian maternal grandmother as a terminal maternal ancestor (TMA) from all administrative provinces of Sicily. After signing an informed consent, each volunteer provided a saliva sample, through mouthwash rinse, and a pedigree chart with known genealogical data. This genealogical information was analyzed to reallocate the samples based on their terminal maternal ancestor (TMA), as follows: Agrigento (*n* = 18), Caltanissetta (*n* = 29), Catania (*n* = 10), Enna (*n* = 5), Messina (*n* = 6), Palermo (*n* = 81), Ragusa (*n* = 8), Siracusa (*n* = 5) and Trapani (*n* = 13), thus representing all the nine administrative provinces of the Island. An anonymous code was assigned to each participant and mitochondrial control region sequence was obtained, as previously described (Modi et al., 2020).

### Population genetics analyses

Genetic structure and differentiation. All mtDNA haplotypes were classified into haplogroups using Haplogrep 2.0 (Weissensteiner et al., 2016) according to the most up-to-date mtDNA phylogeny, as reported in PhyloTree build 17 (http://www.phylotree.org/) (van Oven and Kayser, 2009). The consistency of haplogroup classifications based on control-region and full mitogenome data in Eurasian populations have been confirmed in recent papers (Modi et al., 2020; Cardinali et al., 2022). A diachronic comparison between modern and ancient individuals was achieved through a principal component analysis (PCA) based on haplogroup frequencies (Supplementary Table S5) and conducted by employing the function fviz_pca_biplot from the R-package factoextra (Kassambara and Mundt, 2017).

We also calculated FST values between groups with Arlequin v.3.5 (Excoffier and Lischer, 2010) and visualized them by plotting a heatmap using the function levelplot from the R-package Lattice (Sarkar, 2008). The FST values were also visualized by Multidimensional Scaling (MDS), using the cmdscale function in the R environment.

Phylogenetic inference. The phylogenetic network based on nucleotide variation in the complete mitochondrial genomes, was constructed using the Median-Joining algorithm (Bandelt et al., 1995) implemented in PopART software (http://popart.otago.ac.nz/). We built a Neighbor-Joining phylogenetic trees (Saitou and Nei, 1987) directly on mtDNA sequences using the software MEGA (Tamura et al., 2007) based on pairwise molecular distances corrected using Kimura 2 parameters model (bootstrap value = 500).

Model comparison through Approximate Bayesian Computation. In order to give insights into the origin of Sicanians from Baucina we designed two alternative models of evolution (Continuity and Discontinuity), detailed in Supplementary Table S6, that have been compared through an ABC approach (Supplementary Data for details of the methods and of the demographic models tested). We generated 50,000 simulations under each model, with parameters values drawn from prior distributions (Supplementary Table S6), using fastsimcoal2 (Excoffier et al., 2021) within the ABCToolbox suite (Wegmann et al., 2010). We summarized our data calculating different summary statistics with arlsumstat (Excoffier and Lischer, 2010): number of haplotypes (K), Heterozygosity (H), number of Segregating sites (S), Tajima’s D (TAJIMAD), Fixation index (Fst) and Pairwise differences (Pi). For the model comparison we exploited a machine-learning algorithm named Random-Forest (ABC-RF) (Pudlo et al., 2016). Under the ABC-RF approach the model selection analysis is treated as a classification problem; the classifier is constructed from simulations via the RF algorithm and hence applied to the observed data in order to estimate the posterior probability of the most supported model. To perform the model selection procedure, we used the function abcrf from the R package abcrf, employing a forest of 500 trees (Pudlo et al., 2016). Moreover, following Pudlo et al. (2016), we also included the linear discriminant analysis (LDA) axes as additional summary statistics, enabling the flag LDA = TRUE of the function abcrf. LDA is a method of dimensionality reduction that provides a linear projection of the simulated and observed dataset on a space of dimension M−1 (where M is the number of models tested) called the LDA axes, that optimize the differentiation between the models. The projection of the simulated reference table datasets and of the observed dataset on the first LDA axes gives a visual way to evaluate how much the models are differentiated and if the simulated genetic variation overlaps with the observed variation. Before calculating the posterior probabilities of the most supported model, we computed the confusion matrix and evaluated the out-of-bag classification error.

## Results

### Ancient mitochondrial genomes

We obtained complete or nearly complete mitochondrial sequences from 43 individuals, with an average coverage between 6x and 511x and modern human contamination level lower than 6% for all the samples with the exception of the sample MOK2 that showed a contamination level of 32% (Supplementary Tables S3, S4, Supplementary Data for details). Radiocarbon dating was performed on collagen extracted from petrous bones of individuals from Motya according to the modified-Longin method (Fedi et al., 2007). Radiocarbon dates allowed us to identify samples from two different phases: Bronze Age individuals related to the prehistoric occupation of the island and three individuals from the Phoenician period.

The novel sequences have been deposited in GenBank under accession number: ON496938-ON496980.

### Mitochondrial genetic structure of ancient sicily and pattern of similarities with populations of the mediterranean

For the population genetic analyses we subdivided the newly generated ancient samples into seven groups, considering geographic criteria (i.e., sampling locations) and/or historical and cultural information (i.e. Bronze Age and Phoenician individuals from Motya were treated as two distinct groups and the same was for Sicanian and Greeks from Baucina). Samples that showed a mean coverage <20x and a contamination level >6% were excluded (6 individuals from Baucina and one from Mokarta). A detailed description of the samples analyzed is shown in Table 1.

**TABLE 1:** New samples produced in this study.

Sample id	Mean coverage	Contamination estimates	Locality	Culture/Period	Estimated date	Haplogroup
Bau-11	353.7828	0.02 [0.01–0.03]	Baucina	Sicanian	600–400 BCE	H
Bau-13	66.8664	0.02 [0.01–0.03]	Baucina	Sicanian	600–400 BCE	T2c1d+152
Bau-14	165.0124	0.02 [0.01–0.03]	Baucina	Sicanian	600–400 BCE	J1c
Bau-16	284.8865	0.02 [0.01–0.03]	Baucina	Sicanian	600–400 BCE	T2b3+151
Bau-17	218.338	0.02 [0.01–0.03]	Baucina	Sicanian	600–400 BCE	H1e8
Bau-19	99.3056	0.02 [0.01–0.03]	Baucina	Sicanian	600–400 BCE	T2e7
Bau-1A	458.3061	0.01 [0–0.02]	Baucina	Sicanian	600–400 BCE	I4a
Bau-1	42.5897	0.02 [0.01–0.03]	Baucina	Sicanian	600–400 BCE	H1
Bau-20	346.9911	0.01 [0–0.02]	Baucina	Sicanian	600–400 BCE	T2e7
Bau-22	511.7751	0.02 [0.01–0.03]	Baucina	Sicanian	600–400 BCE	U5a1a1
Bau-23	195.4662	0.01 [0–0.02]	Baucina	Sicanian	600–400 BCE	U5b1d1a
Bau-25	37.4614	0.02 [0.01–0.03]	Baucina	Sicanian	600–400 BCE	H1e8
Bau-26	243.9839	0.02 [0.01–0.03]	Baucina	Sicanian	600–400 BCE	T2b3+151
Bau-4	130.9854	0.01 [0–0.02]	Baucina	Sicanian	600–400 BCE	H
Bau-6	153.5654	0.02 [0.01–0.03]	Baucina	Sicanian	600–400 BCE	T2b3+151
Bau-12G	306.0481	0.05 [0.04–0.06]	Baucina	Sicanian	600–400 BCE	V+72at
Bau-15G	400.9136	0.03 [0.02–0.04]	Baucina	Sicanian	600–400 BCE	T2b3+151
Bau-EST6	186.3743	0.01 [0–0.02]	Baucina	Greek	600–400 BCE	U3a1
Bau-EST7	19.9297	0.06 [0.05–0.07]	Baucina	Greek	600–400 BCE	H+16,311
Bau-EST9	22.924	0.01 [0–0.02]	Baucina	Greek	600–400 BCE	V+72at
MO-101	173.2215	0.01 [0–0.02]	Motya	Phoenician	730–690 cal. BCE	HV0a
MO-103	173.6143	0.01 [0–0.02]	Motya	Bronze Age	1,690–1,495 cal. BCE	H4a1
MO-104a	61.4713	0.01 [0–0.02]	Motya	Bronze Age	2030–1740 cal. BCE	H1j
MO-104b	493.0477	0.03 [0.02–0.04]	Motya	Phoenician	805–535 cal. BCE	HV0+195
MO-106b	499.0567	0.01 [0–0.02]	Motya	Phoenician	755–400 cal. BCE	V
M0-107b	115.0285	0.02 [0.01–0.03]	Motya	Bronze Age	1,640–1,420 cal. BCE	L3b1a5
MO-108	298.649	0.01 [0–0.02]	Motya	Bronze Age	1890–1,620 cal. BCE	I1
MO-109a	82.073	0.01 [0–0.02]	Motya	Bronze Age	1890–1,530 cal. BCE	K2b1
MO-114b	235.6325	0.01 [0–0.02]	Motya	Bronze Age	1,640–1,430 cal. BCE	H4a1
MO-115	141.1135	0.01 [0–0.02]	Motya	Bronze Age	1,610–1,410 cal. BCE	T2h
MO-121a	166.7156	0.02 [0.01–0.03]	Motya	Bronze Age	1775–1,505 cal. BCE	U5b1d1a
MA90	441.5381	0.01 [0–0.02]	Lilibeo	Phoenician	3rd cen. BCE	V25
MOK1	264.0343	0.02 [0.01–0.03]	Mokarta	Late Bronze Age	1,250–1050 BCE	H1
MOK114C	36.0821	0.01 [0–0.02]	Mokarta	Late Bronze Age	1,250–1050 BCE	H5a
T73-7	55.8398	0.02 [0.01–0.03]	Ispica	Bronze Age	1,500–1200 BCE	K1a
T73-M73-1	158.6512	0.01 [0–0.02]	Ispica	Bronze Age	1,500–1200 BCE	X2b+226

We combined the newly produced mitochondrial sequences with data collected from the literature (Lazaridis et al., 2017, 2016; Hofmanová et al., 2016; Lipson et al., 2017; Martiniano et al., 2017; Olivieri et al., 2017; Olalde et al., 2019, 2018; Emery et al., 2018; Fregel et al., 2018; Zalloua et al., 2018; González-Fortes et al., 2019; Villalba-Mouco et al., 2019; Fernandes et al., 2020; Marcus et al., 2020; Rivollat et al., 2020; Skourtanioti et al., 2020; Diroma et al., 2021). We included Sicilian individuals from Fernandes et al. (2020) as other samples from populations of the Mediterranean (Supplementary Table S1). In total we considered 336 individuals from 35 populations, spanning a time range from Neolithic to Iron Age.

**FIGURE 2:**
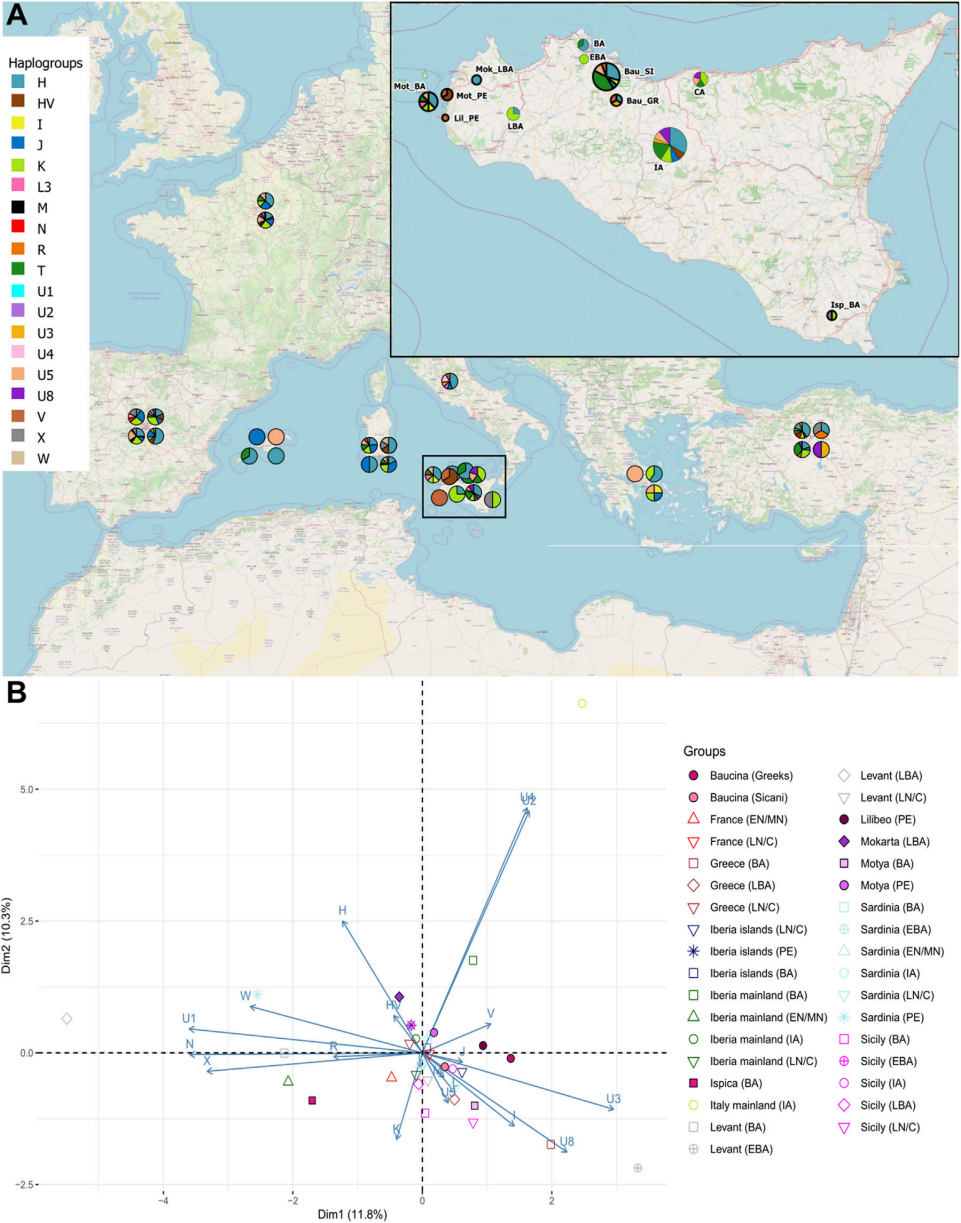
Mitochondrial genetic structure of ancient Sicily. **(A)** Geographical distribution of haplogroup frequency in the analyzed samples. **(B)** Principal Component Analysis (PCA) on the haplogroup frequencies within groups; geographical population are indicated with different colors, while cultures are indicated with different symbols and labelled as follow: EN/MN = Early/Middle Neolithic, LN/C= Late Neolithic/Chalcolithic, BA = Bronze Age, EBA = Early Bronze Age, LBA = Late Bronze Age, IA = Iron Age, PE = Phoenicians.

**FIGURE 3:**
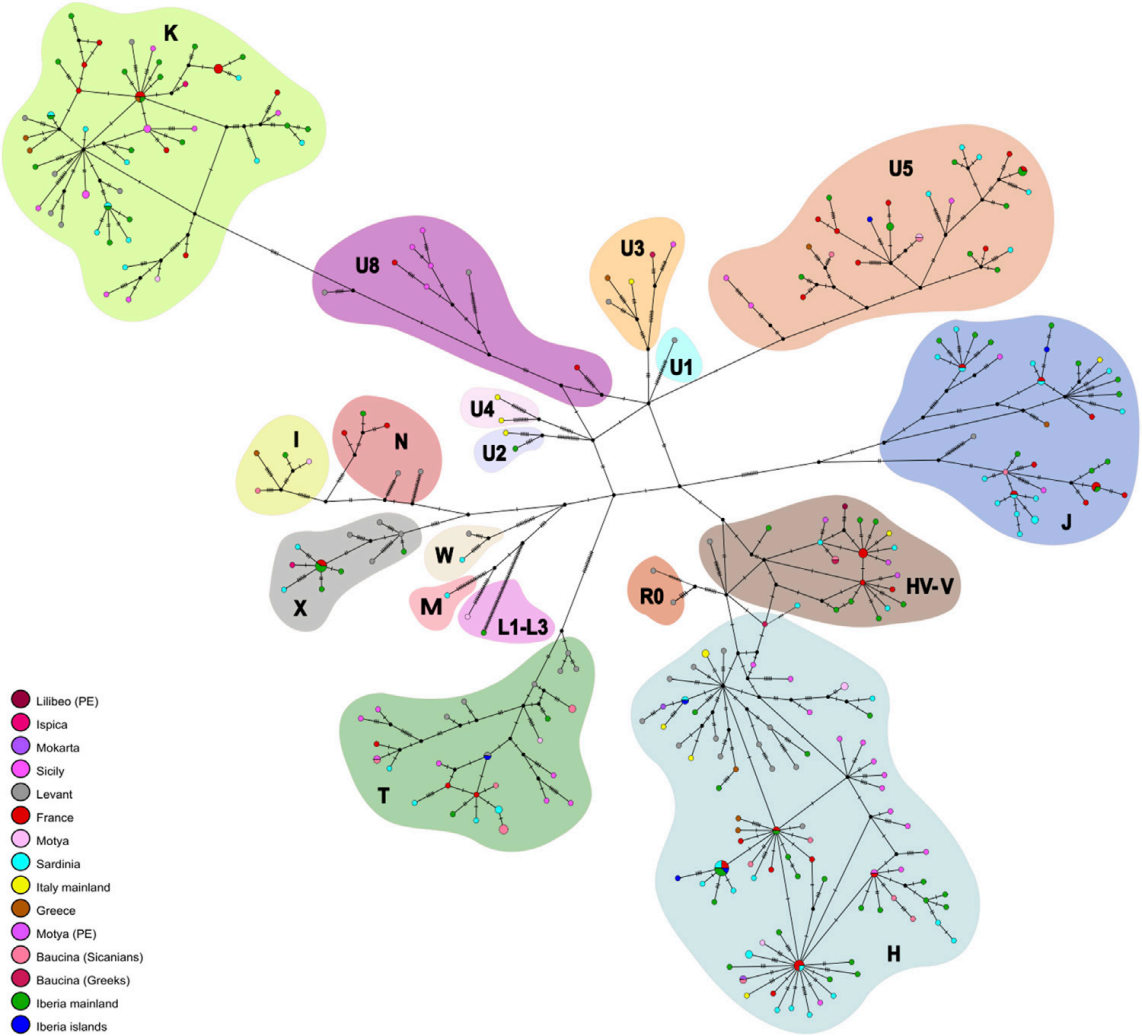
Mitochondrial genetic structure of ancient Sicily. Median joining network. Phylogenetic network based on nucleotide variation in the complete mitochondrial genomes.

Figure 2 panel A shows the distribution of mitochondrial haplogroup frequencies in the whole dataset we built. The haplogroup assignment is also reported in Table 1 and in Supplementary Table S1. In the newly sequenced Sicilian samples, the most frequent haplogroups observed are T and H which reached a frequency of 41% in the Sicanian group from Baucina (*n* = 17) and a frequency of 37,5% in the Motya Bronze Age (*n* = 8) group, respectively. The same haplogroups are represented at high frequencies in previously reported samples from Early Bronze Age Sicily and mainland Iron Age Italy. The Sicilian samples attributed to the Phoenician culture belong to HV and V haplogroups, today found predominantly in the Near East and Caucasus (HV) and Northern Europe (V) (Torroni et al., 2001; Shamoon-Pour et al., 2019). The Bronze Age sample of Motya does not show any HV and V haplogroups, and single individuals reporting T, K, I, and U5. In this group, we also found the presence of a L3 lineage, currently found at high frequency in Northeast Africa and possibly highlighting a genetic link with the African continent during the Bronze Age. The Baucina samples show a variety of haplogroups composition, with Sicanian individuals showing a high percentage of T lineages, followed in frequency by H and U5. The three Greek individuals are assigned to three different haplogroups, H, V, and U3. In the Bronze Age Ispica, we identified one X and 1 K individual. The haplogroups differentiation of the individuals in the dataset is also represented by the Neighbor-Joining (NJ) tree (Supplementary Figure S1), whereas the phylogenetic relationship among haplotypes is represented by the phylogenetic network (Figure 3). The NJ tree (Supplementary Figure S1) reflects the known phylogenetic relationship among mitochondrial haplogroups. Phoenicians in Motya and Lilibeo cluster together, close to Bronze Age and Iron Age samples from Iberian Peninsula, and a Sardinian and an Italian sequence. Sicanians of Baucina and Iron Age indigenous from Polizzello share closeness within the haplogroup T. Bronze Age Motya individuals are spread along the whole tree. The phylogenetic network (Figure 3) describes the genetic relationship among the 336 sequences in the dataset we analyzed. Most of the mitochondrial genomes are unique haplotypes, reported in the network as small circles of a single color. The closeness among sequences in the phylogenetic network mirrors the structure that emerged from the NJ tree.

**FIGURE 4:**
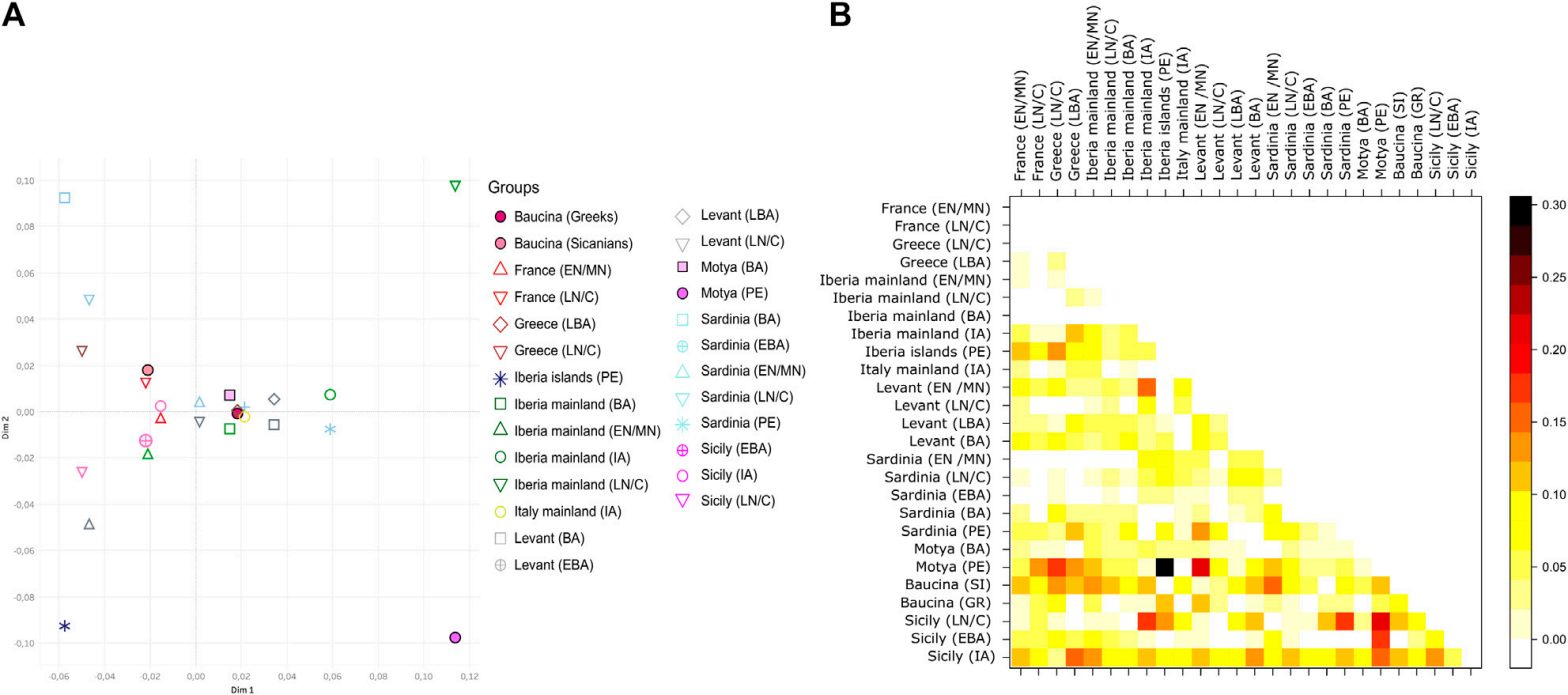
Pattern of similarities with populations of Mediterranean. **(A)** Multidimensional Scaling (MDS) based on a matrix of molecular distances (FST) among groups; geographical population are indicated with different colors, while cultures are indicated with different symbols. **(B)** Heatmap describing a matrix of molecular distances (FST) among groups, each group is indicated by a number. Cultures are labelled as follow: EN/MN = Early/Middle Neolithic, LN/C= Late Neolithic/Chalcolithic, BA = Bronze Age, EBA = Early Bronze Age, LBA = Late Bronze Age, IA = Iron Age, PE = Phoenicians, SI = Sicanians and GR = Greeks.

To better explore the genetic relationship among Sicilian samples we run a Principal Component Analysis (PCA), on the haplogroup frequencies within groups (Figure 2B). In Figure 2B the Sicilian samples are compared with all the Mediterranean populations, from Early Neolithic to Iron Age. With respect to other samples from the Mediterranean Area, Sicilian group occupy a wider range of genetic variation, with the sample from Ispica falling far from other Sicilian individuals, and closer to Bronze Age samples from the Levant and Early Neolithics from mainland Iberia. Phoenicians of Motya and Lilibeo fall closer between each other than with the Bronze Age individuals of Motya. There is no particular closeness with other Phoenicians in Sardinia and Iberia islands. For the Baucina site, the two populations - Sicanians and Greeks - defined through the burial rituals (i.e., structure and design of graves) display genetic differences clearly evident in the PCA (Figure 2B). Moreover, Sicanians fall closer to the Iron Age Sicilian samples from Polizzello, which have also been attributed to the Sicanian culture. The same pattern is evident even taking into account molecular distance among groups instead of haplogroups frequency composition, as shown in the MDS analysis reported in Figure 4A This analysis further highlights the genetic divergence between Sicanians and Greeks in Baucina, with Sicanians falling again close to Iron Age Polizzello, and Baucina Greeks overlapping to the Late Bronze Age Greece and being close to Iron Age mainland Italy and Early Bronze Age Sardinia. The Bronze Age/Iron Age samples fall mainly together in the right portion of the MDS, while Neolithic/Chalcolithic samples occupy the left side of the plot. Exceptions to this pattern are Baucina Sicanians and Iron Age Polizzello, which are closer to Neolithic/Chalcolithic groups. Phoenicians in Motya, Sardinia and Iberia Islands continue to be well separated.

Figure 4 panel B and Supplementary Figure S2 quantify the level of populations’ differentiation due to genetic structure of our dataset. From the heatmap plot of Supplementary Figure S2 it is evident that, among the Sicilian groups, Sicily Late Bronze Age is the most differentiated group, with FST values that reaches 0.5 with Phoenicians in Motya. Sicily Late Bronze Age rather shows genetic similarities with Late Neolithic/Chalcolithic European groups (FST values ranging from 0.07 to 0.12) and in particular with Late Neolithic/Chalcolithic and Bronze Age Greek groups (FST values below 0.05), as also highlighted in Fernandes et al. (2020). To better explore the genetic structure among other groups, we excluded Late Bronze age Sicily from the heatmap, and the resulting picture is shown in Figure 4B. The genetic differentiation among Sicilian groups is higher with respect to that observed in other regions of the Mediterranean. Compared with Sardinia (the other main island of the Mediterranean Sea), the genetic structure within Sicily is stronger, with pairwise populations FST values reaching 0.2–0.3, against a maximum of 0.1 among Sardinian groups. Among the Sicilian groups, the most differentiated sample is Phoenicians in Motya. Interestingly the same group, compared with other populations analyzed, shows the maximum level of genetic differentiation with Phoenicians in the Iberia islands, and, to a lesser extent, with Neolithic/Chalcolithic populations. The most similar group to the Phoenicians in Motya, other than their Bronze Age neighbors, is the Levant Late Bronze Age, with an FST value of 0.011. The Late Neolithic/Chalcolithic group in Sicily also have a high level of genetic differentiation with the other groups, thus highlighting a possible genetic structure since the first colonization by early farmers. The same group shows the lowest level of differentiation with Early/Late Neolithic groups in Sardinia. The Sicanians of Baucina do not show any particular resemblance with populations from Iberian Peninsula, as first postulated by Thucydides (VI, 2, 2); the groups with which they show the lowest differentiation are Neolithic and Late Bronze Age Levant.

**FIGURE 5:**
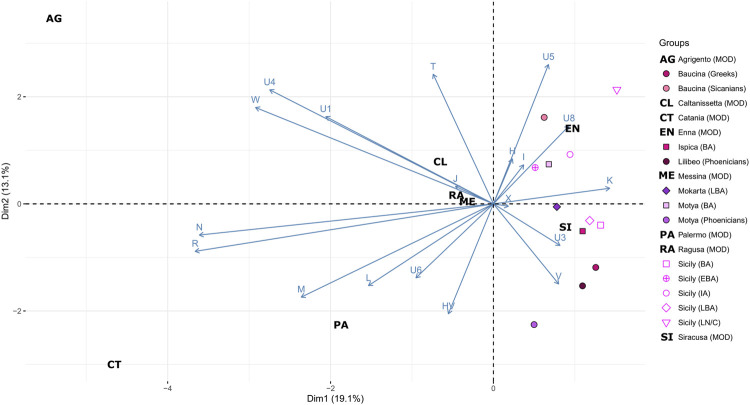
Mitochondrial genetic structure of ancient and modern Sicily. Principal Component Analysis (PCA) on the haplogroup frequencies within Sicilian groups including contemporary populations. Geographical ancient populations are indicated with different colors, while modern populations (MOD) are indicated with letters. Cultural information of the ancient population is represented using distinct symbols and labelled as follow: LN/C= Late Neolithic/Chalcolithic, BA = Bronze Age, EBA = Early Bronze Age, LBA = Late Bronze Age, IA = Iron Age, PE = Phoenicians.

We also compared the ancient mitochondrial genetic structure of Sicily with that present in modern populations of the Island. To this aim, we analyzed the mtDNA haplogroup distribution of a novel dataset of 175 modern individuals from different geographic areas of the region. The most common western Eurasian macro-haplogroups have been identified in modern Sicilians (Supplementary Table S5). The most frequent haplogroup in modern Sicilians is H (34.4%), followed by T (12.6%), L (12.0%), HV (7.4%) and J (6.3%). Haplogroup H is homogeneously distributed throughout the region. Instead, T mtDNAs are absent in the southern part of the region (Ragusa and Siracusa) and very rare (<1.0%) in the north-western provinces (Palermo and Trapani). The high incidence of L mtDNAs in Palermo (23.5%) could be explained by migrations from northern Africa in rather recent historical times (Cerezo et al., 2012). This is also confirmed by their absence in virtually all ancient individuals, with the only notable exception of an ancient L3 mtDNA from Motya. In general, the higher number of macro-haplogroups identified in modern individuals than in ancient ones (18 vs. 11; Supplementary Table S5) could be easily explained by their wider geographical origin. We further explored this evidence through a Principal Component Analysis based on haplogroup frequencies (Figure 5). The PCA plot emphasizes the differences between ancient and modern Sicily, with all the modern groups falling quite far from the variation generated by the ancient individuals. Enna and Siracusa show more resemblance with ancient groups, mostly due to an unexpected high incidence of haplogroup K in these two modern populations (20% in both), which should be further explored in a larger dataset.

### Origin of Sicanian culture

Among the ancient populations dwelling in Sicily, the Sicanians are known to have the most controversial origin. Thucydides (VI, 2, 2) claims that they descended from Iberians, while other authors have described Sicanians as Illyrians descendants (Fine, 1985) or developed locally from Neolithic/Bronze Age people (Diodorus Siculus V, 6,1–3). We exploited the inferential framework of Approximate Bayesian Computation to investigate the origin of Sicanians from Baucina and their relationships with other Sicilian groups (details of the approach in Supplementary Data). For this analysis we decided to exclude the late Bronze Age Sicilians samples collected from Fernandes et al. (2020) due to their higher genetic affinities with Neolithic/Chalcolithic Mediterranean groups rather than with coeval Sicilians groups.

**FIGURE 6:**
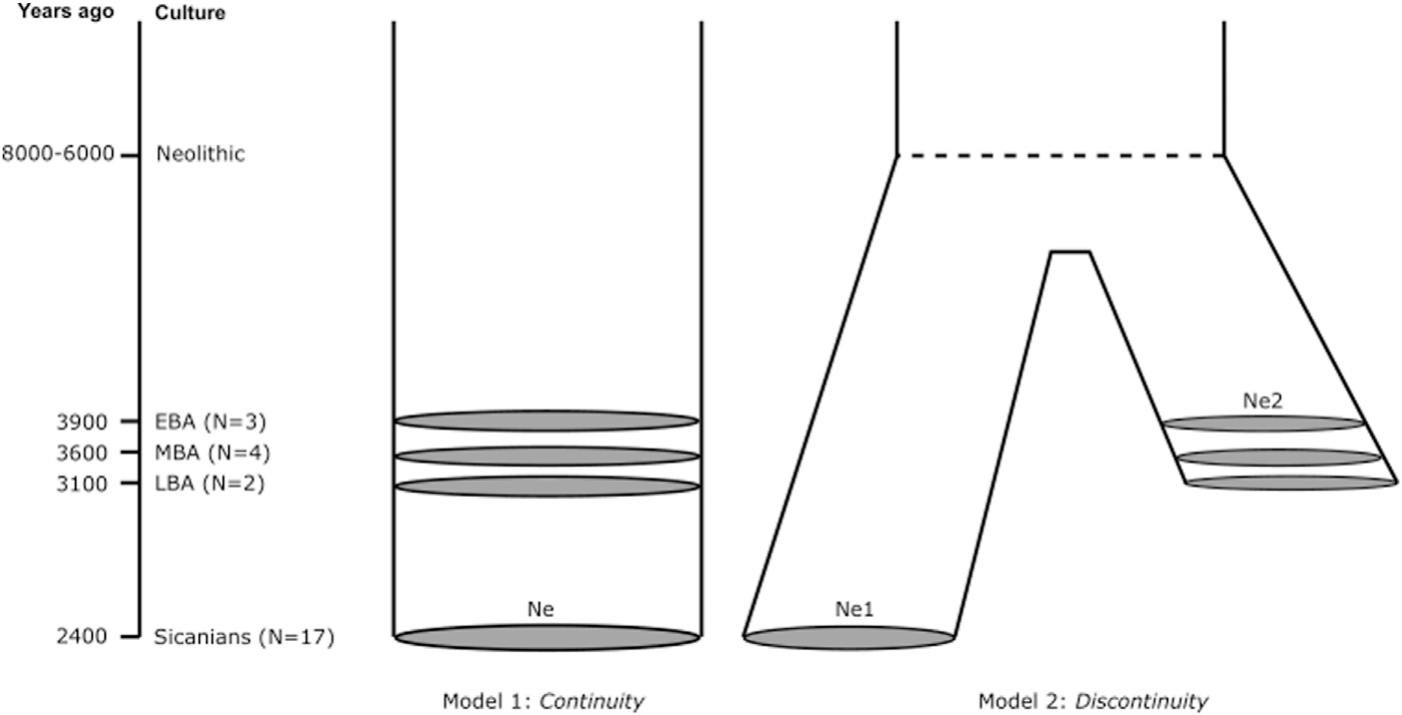
Origin of Sicanian culture. Demographic models describing hypotheses on the origin of Sicanians in Baucina.

We defined two alternative demographic models as shown in Figure 6. The first model, “Continuity”, considers that Sicanians from Baucina are direct descendants of early Bronze Age Sicilian populations, thus assuming a local development of Sicanian culture. The second model, “Discontinuity”, assumed that Sicanians descent from a Mediterranean population that diverged from the ancestral population of Bronze Age Sicilians during the Neolithic (8,000–6,000 BP), thus accounting for an external migration that gave rise to the Sicanian culture in Sicily. We first evaluated the accuracy of our inferential ABC procedure in discriminating between the two models considered, performing a power test computing the confusion matrix and the classification errors. For the Continuity model we obtained a classification error of 30%, while for the Discontinuity model we obtained a classification error of 28%, showing that our procedure is sufficiently able to discriminate between the two models, as also confirmed by the LDA plot (Supplementary Figure S3). We then performed the model selection analysis, which selected the Continuity model as the most supported by the observed data, with a posterior probability of 63%. The results of our ABC analysis are summarized in Table 2.

**TABLE 2:** Model selection. Best model selected (highlighted in bold) by the ABC-RF procedure.

Model	Classification error	Votes	Posterior probability
Continuity	0.3	320	0.63
Discontinuity	0.29	180	

## Discussion

The study of ancient complete mitochondrial genomes from Sicily presented in this paper has generated insight into the diachronic level of population genetic structure of one of the main islands of the Mediterranean Sea. The analysis of mitogenomes from the Mediterranean allowed us to contextualize the Sicilian genetic variation with that of possibly related groups from Early Neolithic to Iron Age. Whereas the comparison with modern Sicilian variation marked the first step towards the understanding the genetic impact of ancient cultures on the modern inhabitants of the Island.

The 36 sequences we analyzed belong to five different archaeological sites, and the haplogroups composition revealed a wide range of variation, even within the same necropolis. The high mitochondrial genetic variation of ancient Sicily is evident also when compared with other ancient European populations, as shown by the PCA in Figure 2B and by MDS plot in Figure 4A. The FST heatmap in Figure 4B further confirms this pattern, highlighting a higher differentiation among Sicilian groups with respect to what is present in other Mediterranean islands (e.g., Sardinia), or in Continental Europe. Indeed, we observed a statistically significant genetic structure within Sicilian groups (AMOVA FST: 0.08947; *p*-value: 0.00139 + −0.00035) whereas in Sardinia, the other main island in the Mediterranean basin, the genetic differentiation is not significant (AMOVA FST: 0.00158; *p*-value:0.42158 + −0.00459). The mitochondrial genetic structure we identify in Sicily is in agreement with the documented historical migrations of populations belonging to different cultures, which made the Island a major Mediterranean crossroad for different populations from Europe, North Africa, and the Levant for a long time (Sarno et al., 2017), and deserves to be further investigated. It also supports that these cultural exchanges were actually accompanied by movements of people, with a possible following exchange of genes.

The eleven individuals sequenced in the necropolis of Motya clustered in two different subgroups, with the three individuals attributed to the Phoenician culture that genetically clustered together and separated from the Bronze Age samples from the same necropolis. These three samples also show similarities with the Phoenician individual from Lilibeo, whose sampling location in the main Island postulates a migration between the two areas or a recent shared ancestry, and further confirms the Phoenician presence within the island of Motya. Another noteworthy pattern of genetic similarities come from the analysis of Baucina and Polizzello samples. In Baucina the individuals have been attributed to the Sicanian culture (26 individuals) and to the Greek culture (3 individuals); the haplogroups compositions and the FST heatmap (Figures 2, 4) actually show the presence of genetic differentiation between these two groups, thus reflecting their different cultural attribution. The observed genetic structure within the Baucina settlement could suggest that a certain social and ethnic distinction was maintained during the Greek colonization. This scenario is also supported by the archaeological records that show a strong Hellenization of the settlements and necropolises starting from the second half of the sixth century BCE, as a consequence of the arrival and establishment of Greeks in the region living alongside the local people (Lyons, 1996; Morgan, 1999).

As highlighted in Figures 2B, 4A, the Sicanians of Baucina show rather more genetic links with the Iron Age samples from Polizzello (Diroma et al., 2021), coming from a necropolis located in the heart of Sicania, and dated ninth-seventh century BCE, than with their Greek neighbors. The genetic closeness of mitochondrial genomes of Sicanians from Baucina and Iron Age indigenous from Polizzello may support the attribution of Polizzello individuals to the Sicanian culture (de Miro, 1988). Additionally, these results reveal a certain genetic homogeneity of the inhabitants of central and western Sicily associated with the same culture.

When ancient Sicilians were contextualized within the Mediterranean domain, we did not find any genetic link between Sicanians individuals (both from Baucina and Iron Age Polizzello) and other Iberian populations. Fernandes et al. (2020) identified Iberia as a key ancestry source for Bronze Age people of Sicily, but the explicit demographic analyses of Sicanian sequences show that this ancestry may not be directly linked to the origin of the Sicanian culture, as originally postulated by Thucydides (VI, 2, 2). The resemblance between Iberia and Sicily seems instead to trace back to Late Neolithic, as emerging also from the low FST values reported in Figure 4B. Our inferential model-based analysis through Approximate Bayesian Computation further supports these results, favoring a local development of Sicanian individuals with a genetic continuity in Central/Western Sicily at least since the Early Bronze Age. Bearing in mind that we are only considering the evolution of the maternal lineage and cannot test other models that may be compatible with the observed genetic variation (such as demographic scenarios that account for sex-biased migrations), our model-based analysis still represents a first step toward a comprehensive and inferential reconstruction of past evolutionary and demographic dynamics in Sicily.

Another interesting similarity pattern came from the Phoenicians in Motya, that showed large resemblance with Levant Late Bronze Age, and the highest FST values (about 0.3) with Phoenicians from Iberian islands. Among the haplogroups identified in the new sequenced samples the most notable is undoubtedly the L3, currently present at high frequency in Northeast Africa (Soares et al., 2012). This L3 sequence found in a Bronze Age individual of Motya explicitly confirmed that the widespread human mobility from North Africa to Europe during the Chalcolithic and Bronze Age, involved also the most remote part of the Island, as emerged also in Fernandes et al. (2020).

Finally, we compared the ancient Sicilian genetic structure with that of modern individuals with known Sicilian ancestry, coming from nine cities around the Island. From the comparison of frequency distribution of mitochondrial haplogroups of ancient and modern Sicilian populations, as well as from the structure emerging from the PCA of Figure 5, we cannot exclude the possibility that Bronze/Iron Age Sicilians made a modest ancestry contribution to modern Sicilians, at least for the maternal lineage. The Y chromosome variation, indeed, has proven to overlap between current and Bronze Age inhabitants of Sicily (Fernandes et al., 2020), postulating a different demographic and evolutionary history for the females and males inhabitants of the Island. A more comprehensive analysis of sex biased processes and of the underlying demographic and evolutionary forces would benefit from an increased and extensive sequencing of modern populations, that would allow to perform explicit comparison between continuity/isolation models from Bronze Age to current time. While our data are indeed consistent with a nearly complete replacement (at least for the mitochondrial lineage) of the pre-Iron Age populations of Sicily by modern inhabitants of the Island, we cannot exclude the hypothesis that locally we may still find a degree of continuity that deserves to be investigated.

This study is restricted to the analysis of a uniparental marker, the mitochondrial genome. Focusing on this marker gave us the opportunity to extend the sampling and the sequencing to a higher number of individuals, so as to adequately representing different cultures dwelling in Sicily in different time periods, and allowing us to identify a structured genetic variation and quantify genetic distances among groups. Albeit limited to the maternal lineage, the present study indeed emphasizes the complex genetic scenario of Sicily since its colonization. The structured genetic variation in culturally defined groups actually supports that cultural processes and exchanges within the Island have been accompanied and promoted by movement of people, and that these dynamics left a footprint on the genetic background of ancient individuals. Modern populations present a rather different pattern of maternal genetic variation; the more homogeneous composition of contemporary uniparental gene pool within Sicily (also reported by Sarno et al., 2014) points towards a recent genetic replacement of pre-Iron Age populations that should be further explicitly addressed.

We acknowledge that the amount of genetic information as well as the inferential power of this uniparental marker is limited with respect to genome-wide ancient DNA data. The analysis of whole-genome variation of different ancient populations from Sicily would provide a more accurate and comprehensive source of information to make inference about past dynamics, such as the time and the origin of principal migration events within the island, the extent of genetic links among contemporary and diachronic groups, and will allow us to explicitly test the hypothesis of a genetic turnover within the island in the last two to three thousand years.

## Data Availability

The datasets presented in this study can be found in online repositories. The names of the repository/repositories and accession number(s) can be found below: https://www.ncbi.nlm.nih.gov/genbank/, ON496938-ON496980, https://www.ncbi.nlm.nih.gov/genbank/, OP081220—OP081394.
