# Supplementary material for: Genetic structure and differentiation from early bronze age in the mediterranean island of sicily: Insights from ancient mitochondrial genomes
Source: Front Genet. 2022 Sep 9;13:945227. doi: 10.3389/fgene.2022.945227 (PMC9500526; doi:10.3389/fgene.2022.945227)
Supplement: Supplementary file 1 [file DataSheet2.PDF]

Supplementary Data

Sampling locations and archaeological context

*Motya***.** Motya, also known as San Pantaleo, is a small island located within the shallow lagoon in the west coast of Sicily, between Marsala and Trapani. Thanks to its geographical location, Motya represented a major berthing place in the sea-routes across the Mediterranean since prehistoric times. The earliest frequentation of the island could go back to the Paleolithic time, as suggested by a bifacial lithic tool recovered in a dry stone wall that delimited an old property of the island. A stable occupation is documented starting from the Early/Middle Bronze Age (17th-12th century BCE) (Falsone et al., 1986; Fresina & Pisano, 1990; Herrmann & Sconzo, 2020; Nigro, 2018), as attested by several archaeological evidence and ceramic finds (Nigro, 2016). During the Bronze Age period, Motya hosted a prosperous indigenous village which continued to leave with several cultural transformations until the end of the 10th century BCE. At the beginning of the 8th century BCE, Motya was colonized by Carthaginian settlers (Nigro & Spagnoli, 2017) and became one of the three main Phoenicians towns in Sicily, as pointed out by the ancient Athenian historian Thucydides. Numerous excavation fields carried out by the Sapienza University, answered questions concerning lifestyle, social organization and agricultural practices of Phoenicians people and the relationships established with the local indigenous population. For the history and archaeology of Motya, see excavation reports by the Missione archeologica a Mozia (MAM) published in the series Quaderni di Archeologia Fenicio-Punica; available online: http://www.lasapienzamozia.it/Bibplio.php (accessed 27 June 2020); see also (Moricca et al., 2021); (Oliveri & Toti, 2020) and references therein. Samples analyzed in the present study were excavated in the area of the "Archaic necropolis" located in the northern coastal strip of the island. Middle Bronze Age tombs, often hosted in caves or pits obtained by regularizing natural cavities, were uncovered in this area. In several cases, prehistoric rock cut tombs "*a grotticella*" were re-used by earliest Phoenicians around 800 BCE, suggesting a targeted choice for the burial place, possibly as remembrance of its prehistoric destination (Nigro & Spagnoli, 2017; Spagnoli, 2008; Vecchio, 2013). Phoenicians and prehistoric burial places, thus, overlapped along the northern bedrock edge of the island. Direct radiocarbon dates allowed us to certainly identify Bronze Age and Phoenicians inhumates.

Samples 104b and 106b refer to the skeletal remains of two individuals belonging to the first generations of Phoenician inhabitants of Motya. This is suggested by stratigraphy, as 104b and 106b were found in secondary burial, re-assembled together with many other human bones, within a sepulchral circular pit in which the remains of some archaic tombs dating back to the 8th century BCE or even earlier (some prehistoric burials were also included) had been collected, in order to make room for new burials (cremation in jars, dating to the 7th century BCE). Subsequently, the city-walls were built over this round structure and, in particular, Tower 4 of the first Motyan wall circuit was erected there. The context of Tower 4, actually underneath Tower 4, is described in Spagnoli, 2008. Human remains of the Phoenician (and prehistoric) burials were often found during the excavation of the city-walls, because the wall circuit, built around 550 BCE, cut through the pre-existing Phoenician and prehistoric cemeteries (Nigro & Spagnoli, 2017). At that time, people buried surely belonged to the élite of the society, and may include members of the first families of Phoenicians (from the Levant and Cyprus) who first settled down on the island. Recent excavations along the city-walls, on the same north-western shore of the island, have revealed in Tower 6 (some 100 m to the west) other burials cut through by the wall structures, which yielded human remains and parts of tomb furnishings, including a monumental inscription of a tomb (Nigro, 2019a). As regards,

sample 101, it was found in between Tower 1 and the later East Tower within Wall M.2 (Nigro, 2019b, 2020), i.e., the earliest city-wall, that means a burial of the first half of 6th century BCE, as also suggested by associated pottery.

Baucina. Baucina is an indigenous site on Monte Falcone (Palermo). An extensive field work conducted by the Superintendency of Palermo in collaboration with the University of Palermo, in 2014, led to the definition of an indigenous Hellenized horizon dating back to the 5th century BCE (Belvedere et al., 2017). The horizon is characterized by different sepulchral types, with multiple burial in caves, or single, in the earth pit, in kalypteres and in enchytrismos. This indicates that at this time indigenous people and people of Greek origin meet at the Baucina site and that, in any case, the site is interested in a significant Greek cultural contribution, such as to influence the funerary ritual behavior.

Mokarta. The site of Mokarta is located on a prominent hilltop (365 m a.s.l.) just a few kilometers from modern-day Salemi (Trapani), in western Sicily.

Archaeological surveys have attested a long period of human occupation, mainly dating from the Early Bronze Age to the Iron Age (Mannino & Spatafora, 1991, 1995; Nicoletti & Tusa, 2012). Due to its strategic position that provides a dominant view of the surrounding areas and key communication routes between Sicily's inland and coast, Mokarta was one of the most important Sicanian settlements in western Sicily. Occupation has been identified at two main locations on the ridge system, Cresta di Gallo and Castello di Mokarta, characterized by a series of distinctive single-room circular huts with pincer-shaped entrance antechambers (Tusa & Nicoletti, 2000). Moreover 61 rock-cut chamber tombs with pottery associated to the cultural facies of Pantalica Nord (1280–1100 BCE) have been documented in the rocky escarpments below these settlements (Mannino and Spatafora, 1995). Archaeological evidence indicates that the settlement was destroyed in a single catastrophic event,and that it was not intensively reoccupied until after the Iron Age (Tusa and Nicoletti, 2000).

Samples analyzed in the present study are dating back to the Late Bronze Age between the 13th and 10th century BCE. The site was probably abandoned around the 10th century BCE following a traumatic event, probably an attack by external populations of the Elymian ethnic group, who at that time settled in the western part of Sicily. The remains of combustion present on the site lead to the reconstruction according to which the attack was followed by a fire. The abandonment of the village was most likely immediate and sudden: as evidenced by the discovery of the skeleton of a young individual (MOK1), presumably trapped in the rubble during the escape, due to the collapse of the roof. The samples MOK2 and MOK114C belong to a multiple burial, dated at the late Bronze Age, but there is no evidence of a particular stratigraphic relationship with MOK1.

*Lilibeo (Marsala)***.** The necropolis occupies about a 90,000 square meter area along the north-eastern side of the town, outside the walls of the ancient Punic-Roman Lilybaeum. The area, bounded to the northwest by the sea, by the outer edge of the Punic moat at the southwest and to the east by the modern church of Santa Maria Della Grotta. It was used from the foundation of the Punic city in the 4th century BCE up to the late imperial age. There was a continuous superimposition of new burials, consequent tampering and readjustments over the centuries.

The monograph by Babette Bechtold (Bechtold et al., 1999) provides a detailed account of the sector of the necropolis investigated between 1987 and 1998. The monograph provides information on areas that were obscured by the superimposition of the modern city on the necropolis. Sample Ma90 derives from Tomb 56 (via Cicerone), dated at 3rd century BCE. The skeleton has been attributed to a young lady (25-30 years old) on the base of skeleton markers.

Ispica. Cava Ispica is a narrow karst valley that extends for about 13 km between Modica and Ispica, in south-estern Sicily. The geomorphology of the cave, the easy defensive position and the proximity to the sea, have helped to make this place one of the largest

settlements in Sicily. Archaeological excavations have testified the human frequentation of the cave at many different stages from the Early Bronze Age to the late Medieval time (Abbate, 2014). The numerous rock dwellings hold traces of human presence until 1693, when Ispica was destroyed by an earthquake. The skeletons here analyzed derive from the Scale Piane necropolis and have been attributed to the Early Bronze Age.

Authentication of ancient DNA data

The authenticity of the consensus sequences was evaluated by analyzing features of aDNA molecules. Ancient DNA, indeed, is typically fragmented into pieces shorter than 100 bp and exhibits miscoding base modifications that accumulate over time, especially at the ends of the fragments (Dabney et al., 2013; Kistler et al., 2017; Sawyer et al., 2012). The most common miscoding lesions are represented by cytosine deamination, that convert cytosine (C) into uracile (U), which code as thymine (T) residues (Briggs et al., 2007). The C-to-T substitution along sequences and fragmentation patterns were evaluated using mapDamage2.0 (Jonsson et al., 2013). Considering the approximately threefold reduction of substitutions observed in partially UDG-treated libraries (Rohland et al., 2015), all the samples showed a deamination rate fully compatible with aDNA degradation (Supplementary Table 3). The average size of fragments ranging from 37 to 63 bp, consistent with ancient molecules (Supplementary Table 3).

Present day human contamination level was assessed using a likelihood-based method implemented in Schumtzi software (Renaud et al., 2015), based on diagnostic sites, which are identified by comparing both consensus sequences to a reference panel of 256 likely contaminants. The incidence of contamination was ≤ 6% for almost all the mitochondrial sequence data (Supplementary Table 4).

Model-based comparison through Approximate Bayesian Computation (ABC)

ABC is a powerful and flexible way to quantitatively compare alternative models and estimating model's parameters. Under these methods, the likelihood functions need not be specified, because posterior distributions can be approximated by simulation, incorporating prior information. The genetic data, both observed and simulated, are summarized by the same set of summary statistics, selected to be informative about the genealogic processes under investigation (for a review see e.g., Bertorelle et al., 2010). In its original formulation, the ABC procedure may require the simulation of millions of data sets of the same size as those observed, thus becoming computationally very expensive as increasing the size of the datasets or the complexity of the models. A new ABC framework has been developed in recent years, based on a machine-learning tool called Random Forest (ABC-RF, Pudlo et al., 2016). Under the RF approach, the model selection stage is rephrased as a classification problem. The machine learning classifier is constructed from the reference table, composed of a set of simulation records made of model indices and summary statistics for the associated simulated data. The reference table serves as a training database for a RF that forecasts model index based on the summary statistics. This classification method has shown to be effective even considering a few thousands simulations, and can accommodate large dimensional summary statistics with no consequences on the estimation performances. Once the classifier is built, it is then applied to the observed data. The posterior probability of the resulting model can be approximated through another RF that regresses the selection error over the statistics used to summarize the data.

One of the most interesting features of ABC is its high flexibility for model checking, i.e., for assessing the quality of the estimates inferred from real data. This is mainly achieved

through the analysis of datasets from the Reference Table, i.e., simulated datasets generated under known conditions, and the estimation of the Classification Error (CE). The CE measures the proportion of time the true demographic history is not correctly identified by the classification algorithm, thus giving a measure of the reliability of the comparison.

In this work we generated data from two alternative evolutionary models, differing from the genealogical relationships among ancient Sicilian populations. Under *Model 1*, we simulated a genetic continuity within the Island since Early Bronze Age, thus assuming that Sicanian culture evolved locally, from an autochthonous group. Under *Model 2*, however, we account for a different genealogical origin of individuals of Sicanian culture, simulating a genetic discontinuity between Bronze Age and Iron Age in Sicily. According to this model, Sicanian individuals derived from a population having a common ancestor with Sicilian groups dating back to Neolithic times. We drew parameters of the demographic models from prior distributions detailed in Supplementary Table 6. We ran 50,000 simulations per model; to perform the model selection procedure, we used the function abcrf from the R package abcrf, employing a forest of 500 trees, a number suggested to provide the best trade-off between computational efficiency and statistical precision (Pudlo et al., 2016). Following Pudlo et al., 2016, we also included the linear discriminant analysis (LDA) axes as additional summary statistics, enabling the flag LDA=TRUE of the function abcrf. The projection of the simulated datasets and of the observed data on the first LDA axes gives us a visual way to evaluate how much the models are differentiated and if the simulated genetic variation overlaps with the observed variation.

References

Abbate, A. (2014). Cava d'Ispica:(1905-2010) oltre cento anni di studi.

Bechtold, B., Frey-Kupper, S., Madella, M., & Brugnone, A. (1999). *La necropoli di Lilybaeum*. L'Erma di Bretschneider.

Belvedere, O., Burgio, A., Bordonaro, G., & Forgia, V. (2017). Baucina (Pa)–Monte Falcone 2014 Indagini nella necropoli. *FOLD&R FastiOnLine Documents & Research*, *380*, 1–7.

Bertorelle, G., Benazzo, A., & Mona, S. (2010). ABC as a flexible framework to estimate demography over space and time: Some cons, many pros. In *Molecular Ecology* (Vol. 19, Issue 13). https://doi.org/10.1111/j.1365-294X.2010.04690.x

Dabney, J., Knapp, M., Glocke, I., Gansauge, M.-T., Weihmann, A., Nickel, B., Valdiosera, C., García, N., Pääbo, S., Arsuaga, J.-L., & Meyer, M. (2013). Complete mitochondrial genome sequence of a Middle Pleistocene cave bear reconstructed from ultrashort DNA fragments. *Proceedings of the National Academy of Sciences*, *110*(39), 15758–15763. https://doi.org/10.1073/pnas.1314445110

Falsone, G., Bonacasa, N., & Buttitta, A. (1986). La scoperta, lo scavo e il contesto archeologico. *La Statua Marmorea Di Mozia e La Scultura Di Stile Severo in Sicilia, Atti Della Giornata Di Studio, Marsala*, *1*, 9–28.

Fresina, A., & Pisano, G. (1990). Saggi di scavo a Mozia. Breve nota preliminare. *Da Mozia a Marsala: Un Crocevia Della Civiltà Mediterranea*, 149–151.

Herrmann, J. T., & Sconzo, P. (2020). Planning Punic cities: geophysical prospection and the built environment at Motya, Sicily. *Antiquity*, *94*(376), 983–998. https://doi.org/10.15184/aqy.2020.97

Kistler, L., Ware, R., Smith, O., Collins, M., & Allaby, R. G. (2017). A new model for ancient DNA decay based on paleogenomic meta-analysis. *Nucleic Acids Research*, *45*(11). https://doi.org/10.1093/nar/gkx361

Mannino, G., & Spatafora, F. (1991). Materiali preistorici dal territorio di Salemi: la Mokarta. *Atti Delle Giornate Internazionali Di Studi Sull'area Elima*, 567–575.

Mannino, G., & Spatafora, F. (1995). *Mokarta: la necropoli di Cresta di Gallo* (Issue 1). Regione siciliana, Assessorato dei beni culturali ed ambientali e della~….

Moricca, C., Nigro, L., Masci, L., Pasta, S., Cappella, F., Spagnoli, F., & Sadori, L. (2021). Cultural landscape and plant use at the Phoenician site of Motya (Western Sicily, Italy) inferred from a disposal pit. *Vegetation History and Archaeobotany*, *30*(6), 815–829. https://doi.org/10.1007/s00334-021-00834-1

Nicoletti, F., & Tusa, S. (2012). L'insediamento del tardo Bronzo di Mokarta (strutture e scavi 1994-97). *L'insediamento Del Tardo Bronzo Di Mokarta (Strutture e Scavi 1994-97)*, 905–916.

Nigro, L. (2018). La Sapienza a Mozia 2010-2016: il primo insediamento fenicio, l'area sacra di Baal e Astarte, il tofet, la necropoli, l'abitato, i nuovi scavi alle mura: una sintesi. *La Sapienza a Mozia 2010-2016: Il Primo Insediamento Fenicio, l'area Sacra Di Baal e Astarte, Il Tofet, La Necropoli, l'abitato, i Nuovi Scavi Alle Mura: Una Sintesi*, 253–277.

Nigro, L. (2019a). Mozia, scavi alle mura (2014-2019). *Mozia, Scavi Alle Mura (2014-2019)*, 21–42.

Nigro, L. (2019b). Qui riposa il «Servo di Melqart». *Archeo 418*, 42–46.

Nigro, L. (2020). Sulle mura di Mozia: stratigrafia e cronologia alla luce dei nuovi scavi della Sapienza (2014-2019). *Sulle Mura Di Mozia: Stratigrafia e Cronologia Alla Luce Dei Nuovi Scavi Della Sapienza (2014-2019)*, 13–64.

Nigro, L., & Spagnoli, F. (2017). *Landing on Motya: The Earliest Phoenician Settlement of the 8th Century BC and the Creation of a West Phoenician Cultural Identity in the Excavations of Sapienza University of Rome: 2012-2016*. Università di Roma" La Sapienza," Missione archeologica a Mozia.

Oliveri, F., & Toti, M. P. (2020). Animals from Motya: Depictions and Archaeological Evidence in the Phoenician Town in Sicily. *Arts*, *9*(3), 96. https://doi.org/10.3390/arts9030096

Pudlo, P., Marin, J. M., Estoup, A., Cornuet, J. M., Gautier, M., & Robert, C. P. (2016). Reliable ABC model choice via random forests. *Bioinformatics*, *32*(6). https://doi.org/10.1093/bioinformatics/btv684

Renaud, G., Slon, V., Duggan, A. T., & Kelso, J. (2015). Schmutzi: estimation of contamination and endogenous mitochondrial consensus calling for ancient DNA. *Genome Biology*, *16*(1), 224. https://doi.org/10.1186/s13059-015-0776-0

Rohland, N., Harney, E., Mallick, S., Nordenfelt, S., & Reich, D. (2015). Partial uracil–DNA–glycosylase treatment for screening of ancient DNA. *Philosophical Transactions of the Royal Society B: Biological Sciences*, *370*(1660), 20130624. https://doi.org/10.1098/rstb.2013.0624

Sawyer, S., Krause, J., Guschanski, K., Savolainen, V., & Pääbo, S. (2012). Temporal patterns of nucleotide misincorporations and DNA fragmentation in ancient DNA. *PLoS ONE*, *7*(3). https://doi.org/10.1371/journal.pone.0034131

Spagnoli, F. (2008). Sepolture intramurali a Mozia. *Sepolture Intramurali a Mozia*, 323–346.

Tusa, S., & Nicoletti, F. (2000). L'epilogo sicano nella Sicilia occidentale: Il caso Mokarta-Capanna 1. *Atti Terze Giornate Internazionali Di Studi Sull'Area Elima, Gibellina, 23-26 Ottobre 1997*, 964–977.

Vecchio, P. (2013). Morte e società a Mozia. Ipotesi preliminari sulla base della documentazione archeologica della necropoli. *Römische Mitt*, *119*, 43–67.
