# Supplementary material for: Genetic structure and differentiation from early bronze age in the mediterranean island of sicily: Insights from ancient mitochondrial genomes
Source: Front Genet. 2022 Sep 9;13:945227. doi: 10.3389/fgene.2022.945227 (PMC9500526; doi:10.3389/fgene.2022.945227)
Supplement: Supplementary file 3 [file DataSheet1.PDF]

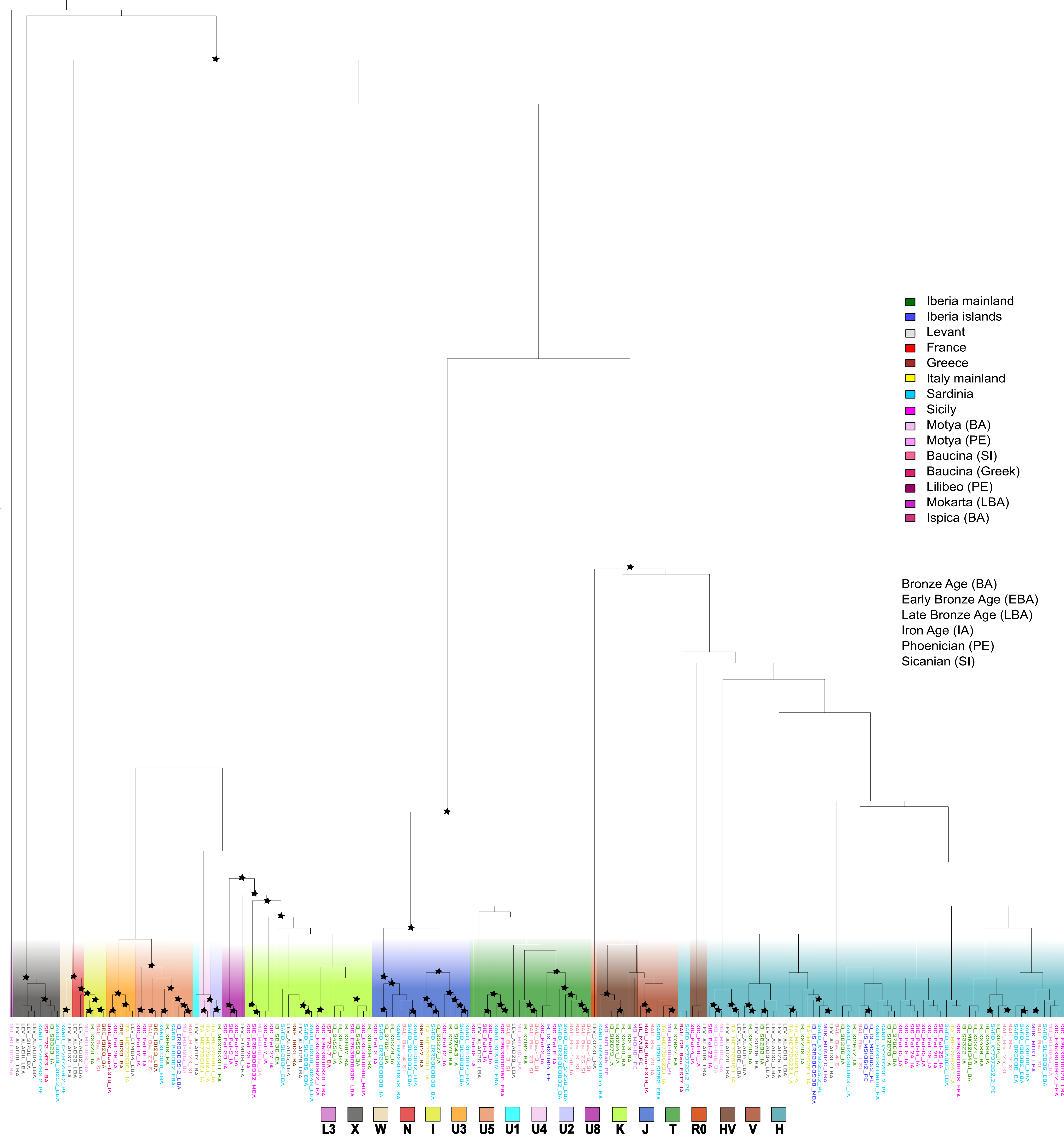
Iberia mainland
Iberia islands
Levant
France
Greece
Italy mainland
Sardinia
Sicily
Motya (BA)
Motya (PE)
Baucina (SI)
Baucina (Greek)
Lilibeo (PE)
Mokarta (LBA)
Ispica (BA)
Bronze Age (BA)
Early Bronze Age (EBA)
Late Bronze Age (LBA)
Iron Age (IA)
Phoenician (PE)
Sicanian (SI)
L3
X
W
N
I
U3
U5
U1
U4
U2
U8
K
J
T
R0
HV
V
H

Supplementary Figure 1. Individual Neighbor-Joining phylogenetic tree. Population and haplogroup membership are indicated by a distinct color, whereas cultural contexts are indicated by different labels. Bootstrap values above 50% are indicated at the nodes by a star symbol.

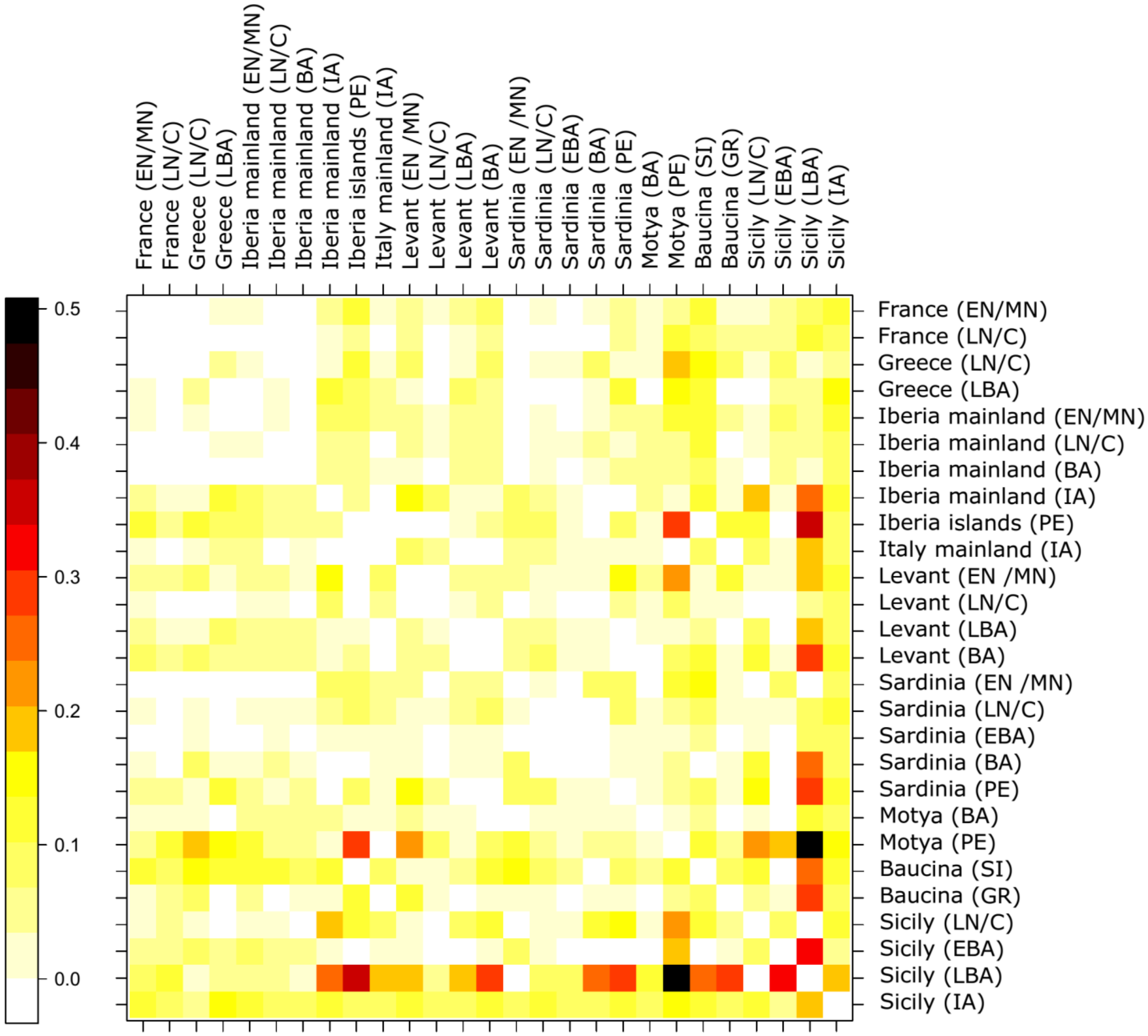

Supplementary Figure 2. Pattern of similarities with populations of Mediterranean. Heatmap describing a matrix of molecular distances (F_{ST}) among groups including Late Bronze Age (LBA) Sicilian group, each group is indicated by a number. Cultures are labelled as follow: EN/MN= Early/Middle Neolithic, LN/C= Late Neolithic/Chalcolithic, BA= Bronze Age, EBA= Early Bronze Age, LBA= Late Bronze Age, IA= Iron Age, PE= Phoenicians, SI= Sicanians and GR= Greeks.

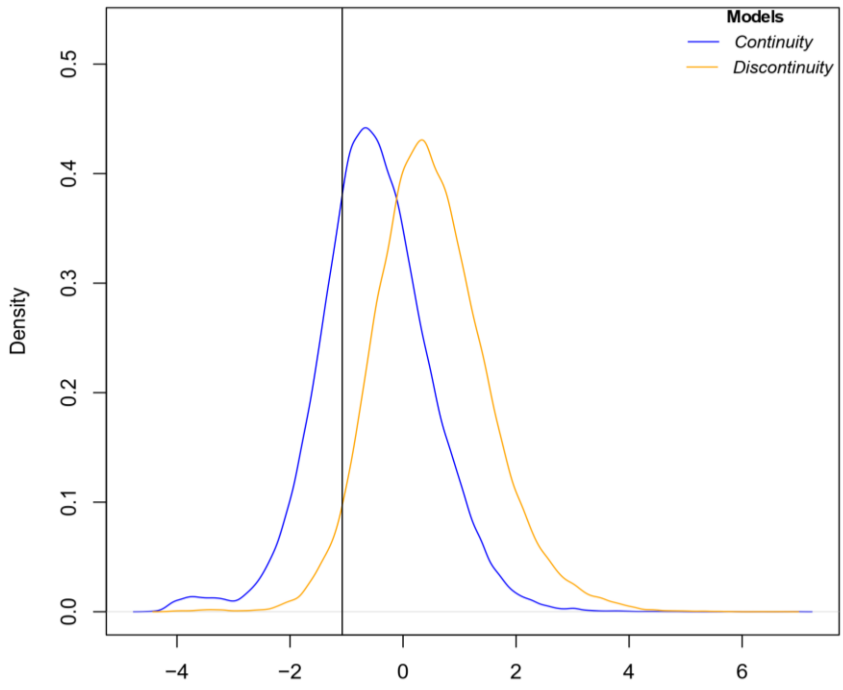

Supplementary Figure 3. LDA plots of the model comparison. The observed data is represented as a straight line.
